# Influence of Periodontal Disease on cardiovascular markers in Diabetes Mellitus patients

**DOI:** 10.1038/s41598-019-52498-7

**Published:** 2019-11-06

**Authors:** Juliana de Fatima Pedroso, Zahra Lotfollahi, Ghadeer Albattarni, Maiara Arrruda Schulz, Andrea Monteiro, Andre Luiz Sehnem, Magnus Ake Gidlund, Antonio Martins Figueiredo Neto, Maria Aparecida Neves Jardini

**Affiliations:** 1 São Paulo State University (UNESP), School of Sciences and Technology, Department of Diagnosis and Surgery, São José dos Campos, Brazil; 2 University of São Paulo (USP), Institute of Physics, São Paulo, Brazil; 3 Labvi Valle, Technique manageress, São José dos Campos, Brazil; 4 University of São Paulo, Institute of Biomedical Science, Department of Immunology, São Paulo, Brazil

**Keywords:** Dental diseases, Diabetes

## Abstract

The objective of the present study was to establish if individuals with Diabetes Mellitus (DM2) and periodontal diseases (gingivitis or periodontitis) presented an increase in the concentration of modified LDL (moLDL) and what is the influence of periodontal treatment on the decrease of moLDL particles with consequent improvement in the parameters of DM2. Twenty-four diabetic patients with periodontitis (Group 1) and twenty-four diabetic patients with gingivitis (Group 2) were followed up for a period of 12 months. Group 1 was treated with periodontal debridement, and Group 2 received supra-gingival scaling and prophylaxis. In both groups, periodontal clinical parameters: probing depth (PD), clinical attachment level (CAL), gingival resection (GR), bleeding on probing index (BOP) and plaque index; inflammatory serum markers (glycemia, A1c, total cholesterol, HDL-cholesterol (HDL-c), LDL-cholesterol (LDL-c), triglycerides and hs-CRP) and oxidized LDL (oxLDL) were measured at baseline, t = 6 and t = 12 months after treatment. Solutions of LDL were analyzed using the nonlinear optical Z-Scan and optical absorption techniques. The periodontal clinical parameters showed significant improvement (p < 0.05) in both Group after 12 months. For both groups, total cholesterol, HDL-c, LDL-c, triglycerides and A1c levels did not show significant reductions after periodontal therapy. hs-CRP levels in Group 1 presented a significant reduction after 12 months. The glycemic rate and the oxLDL concentrations did not show significant differences as a function of time. The optical measurements of LDL solutions revealed an improvement of the LDL-c quality in both groups. Periodontal debridement was able to improve periodontal parameters and the quality of LDL-c in diabetic patients but without changes in the oxLDL concentration in both groups. Considering the clinical relevance, the reduction of infectious and inflammatory sites present in the oral cavity through periodontal therapy may help with the control and prevention of hyperglycemia and precursors of cardiovascular diseases.

## Introduction

Periodontitis is an infection of bacterial origin, characterized by a destructive inflammatory process due to the action of bacteria and their products, and its manifestation comes from the interaction between the causative agent and the host’s immune and inflammatory response^1^.

Diabetes mellitus type 2 (DM2) is a chronic disease in which the pancreas does not produce enough insulin or the body becomes unable to use it effectively^2^. It has a high global prevalence and can affect 439 million people on the planet by the year of 2030^3^.

DM2 is considered as a risk factor for the development of periodontal diseases^4,5^, whereas individuals with periodontitis have a significantly higher prevalence of diabetes when compared with patients without periodontal diseases^6^. Because DM2 and periodontitis present the same inflammatory etiopathogenesis^7^, they demonstrate a bidirectional relationship, as DM2 affects the severity of periodontitis, and this may contribute to the individual’s total inflammatory load, influencing the natural course of DM2^8^.

Inflammatory processes, besides presenting abnormal cytokine production, promote oxidative stress^9^, defined as the severe imbalance between free-radical production and antioxidant defense, leading to possible tissue damage^10^. Thus, the inflammatory responses increase the formation of reactive oxygen species (ROS). Oxidative stress may be the key point in explaining the relationship between DM2 and periodontitis^9^.

In periodontal lesions, the excessive production of ROS is a result of the inflammatory response, which may induce the formation of modified LDL-c (moLDL), mainly oxidized LDL particles (oxLDL) in the blood^11,12^. Studies have shown a positive relationship between the periodontal intervention, performed through periodontal debridement, and the decrease of the serum levels of oxLDL^11,13,14^. It was demonstrated that periodontal treatment was able to cause changes in the serum levels of inflammatory markers, including oxLDL^11^.

It is well established that the presence of moLDL particles in the blood is one of the risk factors for developing cardiovascular diseases (CVD)^15^. These modifications may be due to the presence of ROS in the blood that oxidize these particles. The moLDL particles are no more identified by the cell receptors as a proper cholesterol deliverer and the immunological system will trigger an inflammatory response of the body against these particles. Tocopherols and carotenoids are the endogenous antioxidants present in the LDL particles and protect them against oxidation^16,17^.

Besides the usual biochemical essays employed to identify the presence of moLDL (mainly oxidized) in the blood, the nonlinear optical Z-Scan (ZS) technique has been shown to be useful for evaluating the degree of modification of the LDL particles in the blood—in other words, the “quality” of the LDL particles in the blood^13^. The sensitivity, accuracy and simplicity of the ZS technique, compared to other optical techniques, make it attractive as an effective tool for measuring the sign, magnitude, and order of the nonlinear optical response of materials^18,19^.

In the literature, it is still unclear whether modifications in the level of inflammatory markers and the decrease of oxLDL levels due to periodontal treatment occur in patients with DM2. Therefore, the objective of the present study is to evaluate the quality of the LDL particles from patients with DM2, with generalized periodontitis, compared with those with gingivitis, after periodontal treatment during a one-year follow-up. The ZS technique was used to investigate the characteristic optical response of LDL solution samples, and the UV-visible spectrophotometer was used to measure the linear light absorbance of LDL samples’ solutions.

This multidisciplinary approach broads the horizons of the conclusions of the work, emphasizing the benefits of the dental treatment with respect to the development of CVD in diabetic patients.

## Materials and Methods

This clinical pre-post study with a follow-up of 12 months was registered at clinical trials (NCT03198832-02/06/2017). All procedures received approval from the Human Research Ethics Committee of Institute of Science and Technology (ICT) UNESP in São José dos Campos– São Paulo, Brazil (n^0^1.504.963).

For this study, 356 diabetic patients were screened from May 2016 until August 2017. Forty-eight were included in the pre-established criteria (inclusion and exclusion), of which 24 patients Group 1 had Periodontitis stage III and IV, grade modifiers B and C and 24 patients Group 2 had Gingivitis -dental biofilm - induced mediated by systemic or local risk factors. Considering our previous results^11^(Monteiro *et al*.), a sample size of 22 patients per group would be necessary to detect variations of 0.12 units of moLDL between the groups, with standard deviation 0.14. By using a two-tailed test of variance we have type I error α = 0.05, type II error β = 0.2, and a power of 80%. Twenty-four patients per group were, then, included in the study.

These patients came from the ICT– UNESP (State University of São Paulo Júlio de Mesquita Filho) dentistry school in São José dos Campos – São Paulo, Brazil.

Included in the study were individuals older than 35 years who had DM2 diagnoses for more than five years; had A1c levels between 7% and 11%; and had been diagnosed with periodontitis or gingivitis. They must have presented with the loss of clinical insertions >3 mm in two non-adjacent teeth and the loss of clinical insertions ≥5 mm in 30% or more of the teeth present^20^, and they must have had at least 20 teeth present. They had to agree to participate in the study and sign the informed consent form. Patients with cardiovascular disease, cancer, gastrointestinal disorders, skin diseases, pregnancy, lactation, smoking habit, arthritis, lupus; those who had undergone periodontal treatment within the past 12 months; those who had made use of antioxidant supplements, anti-inflammatories, or antibiotics within the previous three months; those who had changed their glycemic control medication during the past three months; and those with any other diseases of inflammatory origin were excluded.

All of the participants received oral hygiene instructions (Bass brushing technique and the use of dental floss or interproximal brushes), and an experienced clinician (JFP) obtained periodontal parameters. The diabetic patients with periodontitis received full-mouth scaling and root planning with an ultrasonic device (Prof Neo, Dabi Atlante-BR, TipPerio Sub-EVMWQHED3 Ribeirão Preto, SP, Brazil) and manual curettes (Millennium, Golgran, SP Brazil). These procedures were performed under infiltrative local anesthesia with mepivacaine with epinephrine (Mepivalem AD Dentsply Catanduva, SP, Brazil40/0.005 mg/mL) in a single session^21^. Diabetic patients with gingivitis received supra-gingival scaling and prophylaxis in a single session. Every three months, patients from both groups received maintenance therapy through prophylaxis.

Clinical parameters measured were: probing depth (PD), clinical attachment level (CAL), gingival resection (GR), bleeding on probing index (BOP) and plaque index (IPL). A trained examiner (JFP) performed the clinical measurements (Kappa 0.86) using a 15-mm standardized manual periodontal probe (UNC® Hu-Friedy, Jacarepaguá - Rio de Janeiro, Brazil). The examiner’s calibration was done as follows: the examiner measured the PS and the NIC of 10 patients twice in a 24-hour interval. Then the measurements were submitted to the intra class correlation test and the examiner was judged calibrated if a 90% agreement in the measurements was reached. This procedure was repeated until the examiner reached this index.

After anamnesis and the collection of clinical measurements, all patients were referred to the clinical analysis laboratory for the initial blood collection process. Blood was collected into Vacutainer tubes (Vacutainer, BD do Brasil, Curitiba, PR, Brazil) containing ethylenediaminetetraacetic acid from each individual after 12 h of fasting at baseline and then six months and 12 months after the treatment, and the laboratory was blinded to study groups. The first baseline blood collection for laboratory analysis was performed prior to periodontal treatment. The following blood collections (6 and 12 months) were performed 6 and 12 months after the date of periodontal treatment.

The following analysis were: overnight fasting glucose, high-sensitivity C-reactive protein (hs-CRP), glycated hemoglobin (A1c), total cholesterol, HDL-c, LDL-c, triglycerides (TG) and oxidized low-density lipoprotein (oxLDL) level. Plasma was obtained following the centrifugation of the blood at 1000 g for 10 min at 4 °C and was stored at −80 °C. Details about the detection of the oxLDL in the plasma may be encountered in Monteiro *et al*.^11^

### Samples preparation

Blood from patients were centrifuged at 10^3^ g and 4 °C for 15 minutes to isolate the plasma. Benzamidine (2 mM), gentamicin (0.5%) and chloramphenicol (0.25%), PMSF (phenyl–methyl–sulfonyl–fluoride) (0.5 mM) and aprotinin (0.1 unit/mL) were added. The LDL-c (hereafter called the “LDL solution sample” for the optical experiments – ZS and absorbance measurements) was isolated from the infranatant (that contains HDL cholesterol, albumin and other proteins present in the plasma) via sequential preparative ultra-centrifugation^22^ at 10^5^ g and 4 °C using a Hitachi Ultracentrifuge. The protein concentration was determined using a bicinchoninic acid (BCA) protein assay kit (Pierce, Rockford, IL, USA) with bovine serum albumin as the standard.

### Z-Scan technique

The LDL-c sample is illuminated by a laser beam (wavelength λ = 532 nm). Periodic on and off states of about 30 milliseconds of light irradiation are employed. The pulsed-light beam is focused at the position of z = 0 via a lens. All Z-scan experiments were performed at physiological temperature (37 °C). The sample moves along the z-axis, from a position before the focus until a position after the focus. The accuracy of ZS measurements depends on the intensity stability of the laser, the Gaussian profile of the beam, the setup optical alignment and the uniformity of the sample^23^. To avoid spurious effects in our measurements, the laser beam intensity was monitored splitting the beam in two. One of them goes directly to a detector to measure the laser intensity and the other goes to the ZS setup. Eventual fluctuations on the intensity during the experiments are corrected by using the information from the first beam. The second beam enters in the ZS setup and passes through the sample. The transmitted beam is detected by a detector with an iris positioned at its front. At least 10–15 independent measurements (at fixed z position) are made to achieve good data statistics. The reproducibility of our results were checked by measuring the same sample in different days, obtaining results within the experimental errors. More details about the setup, data acquisition and data treatment can be found in our previous works^13,24^. The typical result in the ZS experiment is a peak to valley (or valley to peak) curve of the normalized transmittance as a function of the sample z position. The peak to valley amplitude is proportional to the phase shift (θ) of the thermal lens formed. This parameter depends on the sample absorption coefficient (α) (which is proportional to the concentration of light absorbers), thermo-optical coefficient (dn/dT), laser power (P) and sample thickness (L), and it is inversely proportional to the thermal conductivity (k) of the sample:

1 $$\theta =\frac{0.24P}{k\lambda }\frac{dn}{dT}(1-{e}^{-\alpha L})\cdot $$

In this study, each patient and, correspondingly, each sample had different LDL-c concentrations, so we normalized the values of θ to compare the results from various patients. The parameters of α, P, L and dn/dT were measured independently. Therefore, the ZS allows for the measurement of the sample thermal conductivity, which is a characteristic of the LDL solution.

### UV-vis spectroscopy

The linear-absorbance spectra were obtained via a Uv-vis spectrophotometer with a wavelength range from 200 nm to 1100 nm. Samples were placed into a quartz cuvette with an optical-path length of 1 cm. All experiments were performed at physiological temperature (37 °C). The spectrophotometer measures the extinction spectrum, which is the sum of both the Rayleigh scattering and the absorbance. The Rayleigh scattering is proportional to λ^−4^, and its intensity is estimated for each one of the samples^25^. The absorbance is then calculated removing the scattering contribution from the extinction spectra. As mentioned before, the LDL-c contains various molecules, and each of them has different absorption spectra. For instance, the maximum light absorption of the APOB-100 is at λ_Apo_ ≈ 280 nm^26,27^, the cholesterol at λ_chol_ < 200 nm^28^, the α-Tocopherol at λ_α-Toc_ ≈ 210 nm and the carotenoids at λ_β-Car_ ≈ λ_α-Car_ ≈ 440 and 480 nm^16,17^. In this study, we specifically investigated the absorbance values measured at λ = 480 nm (one of the wavelength at the maximum of absorbance of Carotenoids), at various times of blood extraction, for example, baseline and after six and 12 months.

### Statistical analysis

The data were consolidated and made available on the mean value ± standard deviation (SD) in the case of normal distribution or on the median ± interquartile range otherwise. The evaluation of the normality of the data was made by using the Shapiro-Wilk test. The values of the clinical and laboratorial parameters were compared depending on the case by using: t-test, χ^2^ test, Mann-Whitney Rank Sum Test, one-way repeated measures ANOVA/Tukey Test and Friedman test.

### Ethical approval

All procedures performed in studies involving human participants were in accordance with the ethical standards of the institutional and/or national research committee and with the 1964 Helsinki declaration and its later amendments or comparable ethical standards.

### Informed consent

Informed consent was obtained from all individual participants included in the study.

## Results

All 48 patients completed the 12 months of evaluation. The demographic data of the patients included in the study are described in Table 1. No statistically significant differences were found at baseline among the groups for the parameters of age, gender and number of teeth.

**Table 1:** Clinical and demographic data at baseline of the subjects (mean value ± SD).

Parameters	Group 1 Periodontitis, n = 24	Group 2 Gingivitis, n = 24	p-value (intergroup)
Age (years)	57.6 ± 9.8	56.3 ± 9.5	0.6
Number of teeth	20.75 ± 4.56	25.29 ± 3.06	**0.0001**
Gender (m/f)	12/12	12/12	1^&^
Systolic blood pressure (mm/Hg)	103.4 ± 10.5	102.5 ± 11.3	0.6^*^
Diastolic blood pressure (mm/Hg)	85.0 ± 11.8	79.5 ± 10.5	0.7^*^

Values in bold show statistically significant difference (p < 0.05), *t*-test for independent samples, *Mann-Whitney Rank Sum test, ^&^χ^2^ test.

### Clinical outcomes

The full-mouth periodontal clinical parameters evaluated are presented in Table 2. The analysis of the clinical results obtained showed the mean values of PD, CAL, GR, BOP and IPL for the groups in all times investigated. In Group 1, a significant reduction in PD was found from baseline to t = 6 months and from baseline to t = 12 months (p < 0.0001). The same reduction pattern was observed for CAL, with a significant reduction from t = 0 to t = 6 (p < 0.0001) and from baseline to t = 12 months (p < 0.0001). For BOP, both groups presented a significant reduction from baseline to t = 6 and from baseline to t = 12 months (p < 0.0001) and also showed a significant reduction from t = 6 to t = 12 months (Group 1 p = 0.03 and Group 2 p = 0.02). IPL (in %) both groups showed a significant reduction from baseline to t = 6 (p < 0.0001) and from baseline to t = 12 months (p < 0.0001).

**Table 2:** Full-month clinical parameters (mean value ± SD) at baseline and after 6 and 12 months treatment.

parameter	Time	Group 1 Periodontitis, n = 24	p-value	Group 2 Gingivitis n = 24	p-value
PD (mm)	Baseline 6 months 12 months Reduction (Δ)	3.31 ± 0.80 2.52 ± 0.27 2.57 ± 0.33 0.74 ± 0.68	<**0.0001** <**0.0001**n.s.	2.21 ± 0.25 2.14 ± 0.21 2.04 ± 0.25 0.17 ± 0.27	n.s.= **0.002** n.s.
CAL (mm)	Baseline 6 months 12 months CAL gain (Δ)	3.85 ± 0.93 3.07 ± 0.48 3.12 ± 0.56 0.73 ± 0.68	<**0.0001** <**0.0001**n.s.	2.50 ± 0.51 2.43 ± 0.44 2.33 ± 0.54 0.17 ± 0.27	n.s.=**0.002** n.s.
GR (mm)	Baseline 6 months 12 months	0.55 ± 0.35 0.55 ± 0.35 0.55 ± 0.35	n.s. n.s. n.s.	0.29 ± 0.39 0.29 ± 0.39 0.29 ± 0.39	n.s.n.s.n.s.
BOP (%)	Baseline 6 months 12 months	0.63 ± 0.19 0.23 ± 0.10 0.14 ± 0.07	<**0.0001** <**0.0001=0.03**	0.25 ± 0.14 0.12 ± 0.09 0.06 ± 0.05	<**0.0001** <**0.0001 =0.02**
IPL (%)	Baseline 6 months 12 months	0.81 ± 0.17 0.52 ± 0.20 0.52 ± 0.14	<**0.0001** **0.0001** n.s.	0.77 ± 0.14 0.43 ± 0.21 0.39 ± 0.16	**<0.0001** <**0.0001** n.s.

Values in bold show statistically significant difference (p < 0.05). ANOVA One-way repeated Measures/Tukey Test.

Table 3 shows the pocket stratification data, including moderate and deep pockets, for example, PD = 5 to 6 mm and PD ≥ 7 mm in Group 1. In the analysis of Group 1, it was observed that with respect to moderate pockets, there was a significant reduction in the mean number of them, mean PD (mm), and CAL in all follow-up periods (p = 0.001). The same reduction pattern was observed for deep pockets at t = 6 and t = 12 months.

**Table 3:** Evaluation of moderate and deep pockets at baseline and after 6 and 12 months treatment (mean value ± SD).

parameter	time	Group 1 Periodontitis, n = 24	p-value baseline to 6 months	p-value baseline to 12 months
# of sites with moderate pockets (PD = 5 to 6 mm) per patient	Baseline 6 months 12 months Reduction (Δ)	11.42 ± 5.34 5.25 ± 3.99 4.42 ± 3.86 7.00 ± 4.23	**0.001**	**0.001**
PD of moderate pockets (5 to 6 mm)	Baseline 6 months 12 months Reduction (Δ)	5.35 ± 0.48 3.45 ± 1.25 3.54 ± 1.50 1.81 ± 1.45	**0.001** ^*^	**0.001** ^*^
CAL of moderate pockets (5 to 6 mm)	Baseline 6 months 12 months CAL gain (Δ)	5.37 ± 0.48 4.40 ± 1.29 4.13 ± 1.39 1.24 ± 1.35	**0.001** ^*^	**0.001** ^*^
# of sites with deep pockets (PD ≥ 7 mm) per patient	Baseline 6 months 12 months Reduction (Δ)	4.50 ± 6.50 1.33 ± 1.88 1.21 ± 1.77 3.29 ± 5.70	**0.001** ^*^	**0.001** ^*^
PD of deep pockets (PD ≥ 7 mm)	Baseline 6 months 12 months Reduction (Δ)	7.92 ± 1.57 4.49 ± 1.72 4.49 ± 2.10 3.44 ± 1.94	**<0.0001**	**<0.0001**
CAL of deep pockets (PD ≥ 7 mm)	Baseline 6 months 12 months Reduction (Δ)	8.15 ± 1.51 6.00 ± 1.89 6.07 ± 2.25 2.09 ± 1.97	**0.001** ^*^	**0.001** ^*^

Values in bold show statistically significant difference (p < 0.05). ANOVA One-way repeated Measures/Tukey Test. ^*^Friedman Repeated Measures Analysis of Variance on Ranks.

### Systemic outcomes

In the systemic parameters evaluated (Table 4 and Table 5), glycemia in the Group 1showed statistically significant increase between baseline and t = 6 (p = 0.032).In the Group 2, there is no statistically significant in all follow-up periods.A1cin the Group 1decreases during the first 6 months treatment with statistically significant differences between baseline and t = 6 (p = 0.006). In the Group 2 no statistically significant differences was observed in all follow-up periods. hs-CRP concentrations in the Group 1 presented a decreasing during 12 months treatment and significant difference between baseline and t = 12 (p = 0.041) while in the Group 2 no statistically significant differences was observed in all follow-up periods. The periodontal treatment had no effect on the concentration of oxLDL, HDL-c, LDL-c, total cholesterol and TG in both groups.

**Table 4:** Systemic parameters at baseline and after 6 and 12 months treatment of Group 1- Periodontitis (mean ± SD).

parameter	Before	After treatment				
	Baseline n = 24	6 months n = 24	p-value baseline to 6 months	12 months n = 24	p-value 6 to 12 months	p-value baseline to 12 months
Glucose (mg/dL)	157.7 ± 37.5	182.6 ± 71.4	**0.032**	168.1 ± 48.2	n.s.	n.s.
A1c (%)	9.4 ± 1.7	8.7 ± 1.5	**0.006**	8.9 ± 1.6	n.s.	n.s.
hs-CRP (mg/L)^a^	3.4 ± 4.9	2.2 ± 4.6	n.s.	1.9 ± 3.5	n.s.	**0.041**
Ox-LDL (unit)	1.66 ± 0.60	1.69 ± 0.59	n.s.	1.70 ± 0.55	n.s.	n.s.
Total Cholestrol (mg/dL)	219.7 ± 34.5	220.0 ± 40.0	n.s.	217.2 ± 24.5	n.s.	n.s.
HDL-c (mg/dL)	64.0 ± 18.2	58.5 ± 17.1	n.s.	56.9 ± 15.1	n.s.	n.s.
LDL-c (mg/dL)	114.8 ± 41.7	118.1 ± 41.9	n.s.	125.0 ± 31.0	n.s.	n.s.
TG (mg/dL)	204.2 ± 64.5	216.3 ± 70.3	n.s.	175.9 ± 35.2	n.s.	n.s.
Phase shift: θ	0.10 ± 0.05	0.13 ± 0.06	n.s.	0.16 ± 0.07	n.s.	**0.025**
K (W/m.K)	0.52 ± 0.22	0.38 ± 0.07	**0.004**	0.40 ± 0.08	n.s.	**0.035**
Abs (λ = 480 nm)^b^	0.63 ± 0.23	0.75 ± 0.30	n.s.	0.93 ± 0.33	n.s.	**0.002**

^a^Parameter not normally distributed, expressed as median and interquartile range, Friedman ANOVA. ^b^Abs is LDL-c solution absorbance that is proportional to the sample absorption coefficient α. n.s—not significant. Values in bold show statistically significant difference (p < 0.05). ANOVA One-way repeated Measures/Tukey Test.

**Table 5:** Systemic parameters at baseline and after 6 and 12 months treatment of Group 2-Gingivitis (mean ± SD).

parameter	Before	After treatment				
	Baseline n = 24	6 months n = 24	p-value baseline to 6 months	12 months n = 24	p-value 6 to 12 months	p-value baseline to 12 months
Glucose (mg/dL)	138.1 ± 42.8	122.9 ± 28.4	n.s.	138.5 ± 47.4	n.s.	n.s.
A1c (%)	7.8 ± 1.4	7.3 ± 1.4	n.s.	7.5 ± 1.4	n.s.	n.s.
hs-CRP (mg/L)^a^	2.1 ± 3.6	3.1 ± 3.9	n.s.	2.1 ± 2.7	n.s.	n.s.
oxLDL (unit)	1.80 ± 0.54	1.86 ± 0.82	n.s.	1.75 ± 0.63	n.s.	n.s.
Total Cholestrol (mg/dL)	197.9 ± 29.9	196.2 ± 31.7	n.s.	200.1 ± 22.1	n.s.	n.s.
HDL-c (mg/dL)	61.9 ± 16.6	59.7 ± 14.8	n.s.	58.5 ± 10.0	n.s.	n.s.
LDL-c (mg/dL)	101.5 ± 33.0	99.7 ± 34.8	n.s.	109.7 ± 24.8	n.s.	n.s.
TG (mg/dL)	172.9 ± 60.7	184.2 ± 57.1	n.s.	159.7 ± 33.7	n.s.	n.s.
Phase shift: θ	0.15 ± 0.06	0.19 ± 0.07	n.s.	0.24 ± 0.12	n.s.	**0.0009**
K (W/m.K)	0.46 ± 0.11	0.36 ± 0.04	**<0.0001**	0.34 ± 0.06	n.s.	**<0.0001**
Abs (λ = 480 nm)^b^	1.02 ± 0.38	0.96 ± 0.27	n.s.	1.20 ± 0.47	n.s.	n.s.

^a^Parameter not normally distributed, expressed as median and interquartile range, Friedman ANOVA. ^b^Abs is LDL-c solution absorbance that is proportional to the sample absorption coefficient α. n.s—not significant. Values in bold show statistically significant difference (p < 0.05). ANOVA One-way repeated Measures/Tukey Test.

**Figure 1:**
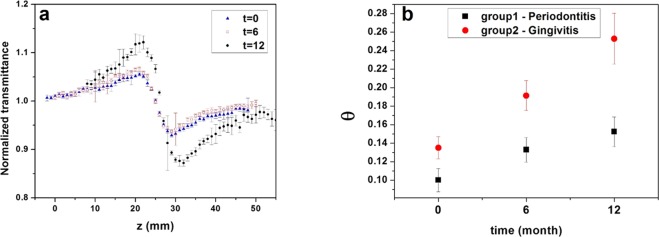
(**a**) Typical Z-scan experimental results of a LDL-c solution sample from a diabetic patient with periodontitis. Normalized transmittance as a function of the sample position, at different times of the periodontal treatment; (**b**) Mean values of the phase shift (θ ± SE) of all patients from the two groups at different times.

The typical results of the ZS experiments of one LDL solution sample of a patient from Group 1, at different times, are shown in Fig. 1a. From the peak to valley amplitude, we obtained the phase shift (θ) (Tables 4, 5 and Fig. 1). In both groups, there are significant difference for phase shift (θ) between baseline and t = 12 months (Group 1, p = 0.025 and Group 2, p = 0.0009). Equation 1 allows for the determination of the thermal conductivity (k) of the LDL solution (Tables 4, 5).

**Figure 2:**
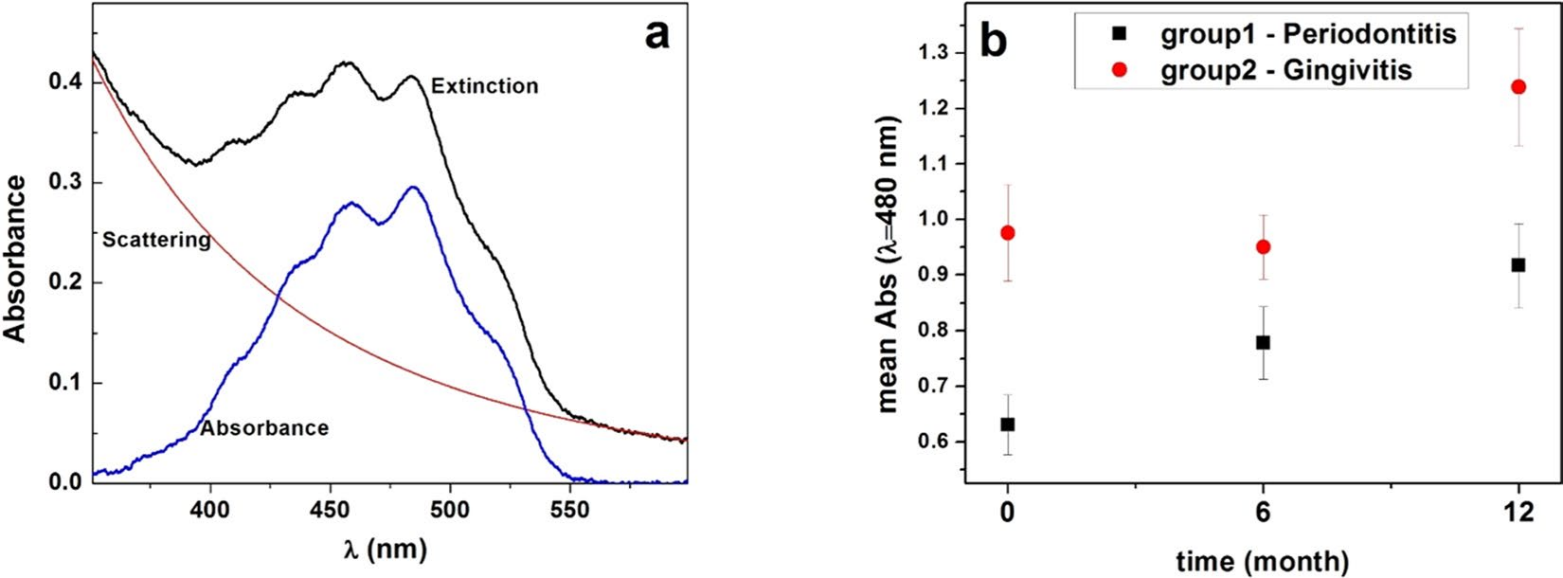
(**a**) Typical absorbance spectrum of a LDL-c sample of a patient from the periodontitis group (Black: Extinction spectrum, Red: Nonlinear Fit (scattering), Blue: Absorbance); (**b**) Mean values of absorbance at λ = 480 nm, in the different times, from the two groups.

The absorbance spectrum of the LDL solution was obtained after the subtraction of the scattering from the extinction spectrum (Fig. 2a). It is worth remarking that it is very similar to the absorbance spectrum of Carotenoids^16,17^. From absorbance spectrum we read its values for 480 nm and a comparison between mean values are shown in Fig. 2b.

## Discussion

Clinical parameters were initially assessed for the full mouth in both groups. Patients with gingivitis had significant reductions in BOP and IPL after six and 12 months of the beginning of treatment. In Group 1, there were significant reductions in PD and CAL, confirming the efficacy of the treatment. The analysis of Group 1 with respect to the presence of moderate and deep pockets, the choice of periodontal debridement in a single session^29^, showed that PD ≥ 7 mm experienced a statistically significant reduction after treatment (p < 0.0001).

*In vitro* ZS experiments, where native human LDL particles were oxidized with CuSO_4_ as a function of the time, showed that the nonlinear optical responses of the samples depend on the oxidative degree of the LDL particles^24^. The higher the oxidative degree of the particles, the lower the nonlinear optical response measured by the ZS technique. Similar results were obtained with LDL particles modified by glycation in *in vitro* experiments^25^. Solutions with LDL particles modified by glycation present a smaller nonlinear optical response with respect to solutions with native LDL. In a previous work^13^, we investigated the quality of the LDL in the blood of patients with periodontitis before and after the periodontal treatment. Before the treatment, the ZS results showed that the LDL particles of these patients were heavily modified. After 12 months of the periodontal treatment, the ZS results showed characteristics of more healthy particles. This conclusion was supported by complementary analysis showing that periodontal treatment modifies the level of several inflammatory markers that, in principle, reduces the risk of CVD.

We showed that it is possible to detect minimum modifications in the LDL particles with the ZS, which is not detectable with other techniques^25,30,31^. The LDL particles feature various components, such as phospholipids, APOB-100, carotenoids, cholesterol esters, triglycerides and free cholesterol. Each of them contributes differently to the sample thermal-lens optical response^32^. The LDL components absorb the light from the laser and transform it into heat, which propagates across the sample. In particular, the carotenoids present a linear light-absorption maximum at wavelengths of about 480 nm.

In both groups investigated, we saw an improvement of the LDL quality due to the 12 months dental treatment (θ increases), and the values of θ from the patients with periodontitis were always lower when compared with those from the gingivitis group. Comparing both groups, the differences between the θ mean values increase with time, faster in the case of the gingivitis group. More interesting, even after 12 months of the periodontal treatment, the quality of the LDL from patients with periodontitis did not reach the level of that of the gingivitis.

These results indicate that the periodontitis left a sequel in the patients, even after the treatment, with respect to the quality of the LDL particles present in their blood. The increasing mean values of θ with the time of dental treatment may have two origins. One is the possible increasing sample-absorbance values due to the increasing number of antioxidants in the LDL-c. The other possibility is the decreasing values of the thermal conductivity of the solution due to the decreasing amount of oxidation products in the plasma and consequently in the LDL solution investigated.

The thermal conductivity, k, is calculated directly from the measurement of θ as remarked earlier (Eq. 1). The mean value of k from both groups decreased with time. Moreover, the mean values in Group 1 were higher than those in Group 2 at the same time. Between baseline and t = 12 months, the mean values of k in Group 1 and Group 2 were significantly different, with p = 0.035 and p < 0.0001, respectively. Since the oxidation process form lipid hydro peroxides, these oxidation products were shown to increase thermal conductivity^24^. In this framework, we expected that the concentration of antioxidants in the LDL particles were inversely proportional to k.

In both groups the absorbance at λ = 480 nm (Fig. 2b) increased after 12 months treatment. The mean values of absorbance in Group 2 were always bigger than those in Group 1. For Group 1, there were significant differences in the mean values of absorbance between baseline and t = 12 months (p = 0.002). Assuming that the absorbance at λ = 480 nm was (mainly) proportional to the concentration of carotenoids, we can conclude that: i) the number of carotenoids in Group 2 was higher than that of Group 1 at the same time, and ii) patients from both groups showed an increase of the number of carotenoids in their LDL particles as a function of time.

The LDL particle’s core consists of triglycerides, esterified and free cholesterol molecules. Its surface is a monolayer comprising phospholipids, free cholesterol, α-tocopherol, carotenoids, and the APOB-100^33^. Carotenoids are hydrophobic molecules localized in the lipid membrane and their orientation towards lipids depends on their structural features and polarity^34^.

In the ZS experiment, light is mainly absorbed by the external surface layer of the LDL particle, i.e., the phospholipid heads, APOB-100, α-tocopherol, carotenoids and cholesterol present in this layer. APOB-100 and α-tocopherol present maximum absorbance in wavelengths about 280 nm and 210 nm respectively, and in 532 nm their light absorptions are small^16,17,26,27^. The absorption spectra of most carotenoid exhibit maximum absorption around 400–500 nm depending on the solvent they are diluted in^35^. Some carotenoids (e.g., β-carotene), in the presence of phospholipids, have absorption spectrum broadening, presenting a non-negligible absorbance in 532 nm^36^. The phospholipids and free cholesterol present in the surface layer of the LDL have also small absorptions in 532 nm, comparing to that of carotenoids^28,32^. Therefore, LDL’s phospholipids, APOB-100, α-tocopherol, and cholesterol contributions to the formation of the thermal-lens are small.

The absorbance spectrum of the LDL (Fig. 2a) is the result of the absorption of all the molecules present in the phospholipid layer and the APOB-100. Consequently, we expect that the main contribution to the formation of the thermal-lens is the light absorption by the carotenoids present in the LDL. We cannot, however, claim that the carotenoids are the unique responsible for the thermal-lens formation, since a small contribution from other molecules exist. This fact is a limitation of our analysis.

## Conclusions

Periodontal debridement was able to improve periodontal parameters in diabetic patients with periodontitis, and the BOP and IPL patterns in patients with gingivitis, but without changes in the oxLDL concentration in both groups. However even after one-year of periodontal treatment, the quality of the LDL particles in patients of Group 1 did not reach that of Group 2. Measurements of the thermal conductivity and optical absorbance of LDL solutions revealed an improvement of the LDL quality in both groups with the treatment. The physical parameters of θ, k and Abs were shown to be more adequate for determining the quality of the LDL particles of patients, which is important for evaluating their CVD risk.
